# Trait Respect Is Linked to Reduced Gray Matter Volume in the Anterior Temporal Lobe

**DOI:** 10.3389/fnhum.2020.00344

**Published:** 2020-08-25

**Authors:** Hironori Nakatani, Yulri Nonaka, Sera Muto, Michiko Asano, Tomomi Fujimura, Tomoya Nakai, Kazuo Okanoya

**Affiliations:** ^1^Department of Information Media Technology, School of Information and Telecommunication Engineering, Tokai University, Tokyo, Japan; ^2^Graduate School of Arts and Sciences, The University of Tokyo, Tokyo, Japan; ^3^RIKEN Center for Brain Science, Wako, Japan; ^4^Center for Institutional Research, Educational Development, and Learning Support, Ochanomizu University, Tokyo, Japan; ^5^Human Informatics Research Institute, National Institute of Advanced Industrial Science and Technology, Tsukuba, Japan; ^6^Center for Information and Neural Networks (CiNet), National Institute of Information and Communications Technology (NICT), Suita, Japan; ^7^Graduate School of Frontier Biosciences, Osaka University, Suita, Japan

**Keywords:** social emotion, trait empathy, empathic concern, magnetic resonance imaging, voxel-based morphometry, sonkei (a Japanese concept of respect)

## Abstract

Respect is a positive other-oriented social emotion upon the recognition of excellence in others. We previously reported that respect-related brain activity in the left anterior temporal lobe (ATL). Since brain activity and structure are often involved in common cognitive functions, we investigated the morphological properties of the left ATL using voxel-based morphometry analysis. We found an association of trait respect with reduced gray matter volume (GMV) in part of the left ATL. Moreover, since the ATL is involved in general conceptual knowledge, we investigated the relationships between other social emotions with similar properties as respect and the GMV of the left ATL. We observed an association of reduced GMV with empathic concern, which is an other-oriented and affective aspect of trait empathy. Our findings indicated an association of the left ATL with other-oriented and affective aspect of social emotions.

## Introduction

We often experience respect upon genuine recognition of excellent behavior, achievement, moral qualities, and personalities in other persons (Kitayama et al., 2006; Li and Fischer, 2007; Muto, 2014, 2016a,b,c). Respect is important for self-development and interpersonal relationships. For example, it strongly motivates good behavioral aspects and contributes to the acquisition of the good qualities that the respected person possesses; further, it enhances interaction with respected persons (Li and Fischer, 2007).

Respect consists of ought and affect aspects (Li and Fischer, 2007). The ought aspect of respect refers to an attitude or disposition toward a particular person based on political, moral, and legal considerations. It is linked to a right-based moral principle without variations according to temporal and contextual particularities (Li and Fischer, 2007). The affect aspect of respect is mostly emotional and is generated from a specific social context or relationship (Li and Fischer, 2007). It is among the positive other-praising emotions involving admiration, awe, reverence, and elevation that are associated with appreciating or praising other persons (Haidt, 2003; Algoe and Haidt, 2009; Sweetman et al., 2013). The present study aimed to investigate the affect aspect of respect.

As all participants in this study were Japanese, we designed this study based on the Japanese concept for respect, *sonkei* (Muto, 2014, 2016a,b,c). Muto (2016b) investigated a Japanese concept reflecting trait respect which refers to one’s proneness to experience respect toward others. Trait respect is comprised of three factors: warm respect, worship, and awe, which are positively and moderately intercorrelated but differentially associated with the Big Five personality traits. Warm respect is a feeling of liking and looking up to the worth or excellence of a person who is considered superior and is positively associated with Extraversion and Agreeableness. Worship refers to the excessive or blind adoration of a person who is considered superior and is positively correlated with Neuroticism and Openness to Experience. Awe is an overwhelming feeling of reverence produced by a person who is considered superior. It is positively related to Neuroticism and negatively related to Extraversion, Conscientiousness, and Openness to Experience. As well as trait respect, the emotion concept of respect has several categories (Muto, 2014). Previously, we conducted a functional magnetic resonance imaging (MRI) study (Nakatani et al., 2019) on the neural substrates of respect using three subordinate categories: warm respect, worship, and awe. We observed respect-related brain activity in the left anterior temporal lobe (ATL).

Based on these previous findings (Nakatani et al., 2019), we raised two questions. The first question was whether the structure of the left ATL is associated with respect since brain activity and structure are often involved in common cognitive functions (Maguire et al., 1999, 2000; Spiers and Maguire, 2006). The second question was regarding the relationship between the morphological properties of the left ATL and other social emotion categories. The ATL is involved in general conceptual knowledge (Rogers et al., 2004; Olson et al., 2007, 2013; Patterson et al., 2007; Zahn et al., 2007, 2009; Wong and Gallate, 2012); therefore, ATL activity is not specific to any social emotion category and their valence (Zahn et al., 2007, 2009). Given the association between brain activity and structure, we speculated that the ATL structure is associated with other social emotions with similar properties as respect.

To address these questions, we aimed to analyze the relationship between trait respect and gray matter volume (GMV) of the left ATL using voxel-based morphometry (VBM). The semantic structure of respect in Japanese is almost the same across generations (Muto, 2016c). As we were interested in the common properties of trait respect-related brain structure among generations, we investigated participants with a wide age range. Further, since trait respect is comprised of three subordinate categories, we aimed to analyze the detailed GMV properties with respect to these subordinate categories, and subsequently analyze the relationship between the GMV of the left ATL and trait empathy. Trait empathy is comprised of four factors with each factor being characterized by two parameters. The parameters are cognitive/affective and self-/other-oriented aspects (Davis, 1980, 1983; Decety and Jackson, 2004; Banissy et al., 2012). An association of the morphological properties of the left ATL with other social emotion categories with similar properties as respect would indicate an association of the other-oriented and affective aspects of trait empathy with the GMV of the left ATL. Further, we aimed to analyze the relationships among trait respect, trait empathy, and the GMV using a conditional process analysis (Hayes, 2013; Muller Prado et al., 2014) in order to determine the direct and indirect effects of trait respect and trait empathy on the GMV.

## Materials and Methods

### Participants

We enrolled 142 participants with a wide age range (83 men and 59 women: mean age = 38.7 years, standard deviation = 14.6 years, range = 18–74 years). All the study participants were Japanese who provided written informed consent.

The Research Ethics Committee of the Graduate School of Arts and Sciences of the University of Tokyo approved the study procedure. All experiments were performed in accordance with guidelines regarding this approval.

### Experimental Procedure

The study consisted of two parts: questionnaire surveys and MRI scanning. Questionnaire surveys were conducted immediately before and after MRI scanning to evaluate trait respect and trait empathy.

### Questionnaire

We used the Trait Respect-Related Emotions Scale (TRRES) (Muto, 2016b) to evaluate trait respect. The TRRES was developed based on the Japanese concept of respect, *sonkei*. The scale includes 35 items regarding several aspects of trait respect, which is comprised of three subordinate categories: warm respect, worship, and awe. The participants answered each item using a 7-point Likert scale (1: definitely not to 7: definitely yes). We calculated the score for warm respect, worship, and awe, respectively. Test score reliability was assessed using Cronbach’s alpha. In this study, the values of Cronbach’s alpha of warm respect, worship, and awe were 0.927, 0.875, and 0.860, respectively. The score for trait respect was evaluated with the average score of three subscales.

The Interpersonal Reactivity Index (IRI) is a multi-dimensional scale of trait empathy comprised of four subordinate categories: perspective-taking, fantasy, empathic concern, and personal distress (Davis, 1980). Perspective-taking and fantasy are cognitive aspects of empathy while empathic concern and personal distress are affective aspects of empathy. These categories are defined as follows (Davis, 1983). Perspective-taking refers to the other-oriented tendency of spontaneously adopting the psychological point of view of others. Fantasy refers to a self-oriented tendency of transposing oneself imaginatively into the feelings and actions of fictitious characters in books, movies, and plays. Empathic concern refers to other-oriented feelings of sympathy and concern for others who are unfortunate. Personal distress refers to self-oriented feelings of personal anxiety and unease, as well as intense interpersonal settings. We used the Japanese version of the IRI to assess trait empathy (Himichi et al., 2017). This was a 28-item scale where participants answered each item using a 5-point Likert scale (1: definitely not to 5: definitely yes). In this study, the values of Cronbach’s alpha of perspective-taking, fantasy, empathic concern, and personal distress were 0.659, 0.778, 0.755, and 0.808, respectively.

### MRI

Brain structures were assessed using a 3T whole-body MRI system (MAGNETOM Prisma; Siemens, Erlangen, Germany) with a 64-channel head coil. The participants were asked to refrain from head movement with their heads being immobilized using cushions in the head coil. We acquired whole brain high-resolution T1-weighted anatomical images (1 × 1 × 1 mm^3^) using a magnetization-prepared rapid acquisition gradient-echo sequence (TR = 2000 ms; TE = 2.9 ms; FA = 9 degrees; FOV volume = 256 × 256 × 256 mm^3^).

### Voxel-Based Morphometry

A VBM analysis was performed using the SPM12 software (Wellcome Trust for Neuroimaging, Institute of Neurology, University College, London, United Kingdom) since it allows voxel-wise GMV analysis (Ashburner and Friston, 2000). Before VBM analysis, all anatomical images were manually coregistered using the standard T1-weighted anatomical template provided by SPM12 where the anterior commissure is the origin of the three-dimensional Montreal Neurological Institute (MNI) coordinate system. Coregistered anatomical images were segmented into three tissue classes: gray matter, white matter, and cerebrospinal fluid (Ashburner and Friston, 2005). Subsequently, a diffeomorphic anatomical registration through exponentiated lie algebra (DARTEL) (Ashburner, 2007) was applied for spatial normalization and accurate inter-participant registration of anatomical images. The DARTEL generated a population-specific template obtained as an among-participant average of anatomical images. Next, anatomical images were spatially normalized to the MNI coordinate system. The modulation was applied to preserve the GMV within each voxel. Here, the spatially normalized gray matter was multiplied by its relative volume (Ashburner and Friston, 2000). The modulated anatomical images regarding GMV were resampled to 1.5 × 1.5 × 1.5 mm^3^ and smoothed using an 8-mm full width at half-maximum Gaussian kernel.

### Region of Interest and Small Volume Correction Analysis for Trait Respect

Since this study focused on the left ATL structure based on our previous findings of an association of its activity with respect (Nakatani et al., 2019), we defined the anatomical region of interest (ROI) in the left ATL for small volume correction (SVC) analysis (Poldrack, 2007).

First, we defined an anatomical ROI in the left ATL using Brainnetome Atlas^1^. Since the ATL is located in Brodmann 20 and 38 (Zahn et al., 2007; Wong and Gallate, 2012), we employed the four atlas files in these regions, which include the medial and lateral parts of the temporal cortex in the left hemisphere. Since the atlas files represent the probability maps of brain regions, they were binarized using a threshold of 0.5. We defined the ROI in the left ATL by combining the binarized atlas files.

Subsequently, we used a general linear model (GLM) to identify brain regions associated with trait respect. The GLM included the score of the trait respect as a regressor with age, sex, and whole brain volume as confounding variables. To evaluate the statistical significance of the relationship between trait respect and the GMV in the ATL, we restricted the region for analysis to the anatomical ROI in the left ATL using the WFU Pick Atlas software (ANSIR Laboratory, Wake Forest University School of Medicine, United States) and applied SVC with voxel-based family wise error (FWE) correction. Statistical significance threshold was set at *p* < 0.05.

Finally, as trait respect is comprised of three subordinate categories: warm respect, worship, and awe, we also evaluated relationships between each subordinate category and the GMV in the ATL.

### Region of Interest and Small Volume Correction Analysis for Trait Empathy

After identifying brain regions associated with trait respect, we used GLM to analyze the relationships between the region and each subordinate category of trait empathy. In this analysis, the ROI was the trait respect-related region. The GLM adjusted for age, sex, and whole brain volume as potentially confounding variables. To evaluate the statistical significance of the relationship between the region and each subordinate category of trait empathy, we applied SVC within the ROI. Statistical significance threshold was set at *p* < 0.05.

### Mediation Analysis

To assess the direct and indirect effects of respect and empathy on the GMV of the region associated with trait respect, we conducted a mediation analysis (Hayes, 2013; Muller Prado et al., 2014). We evaluated whether empathy and respect mediated each other’s effect on the GMV. When we identified brain regions associated with trait respect, the GMV of each voxel was adjusted for age, sex, and whole brain volume as potentially confounding variables. Thus, in this mediation analysis, we also adjusted the GMV of trait respect-related regions for age, sex, and whole brain volume. We used the bootstrap method to calculate the 95% confidence intervals of the estimated parameters and evaluated statistical significance of them. A total of 5,000 bootstrap replications were carried out.

## Results

**FIGURE 1:**
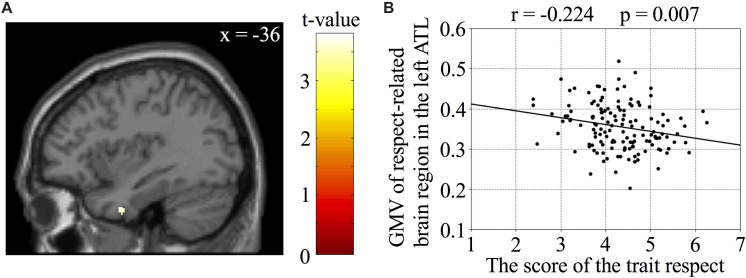
A region in the left anterior temporal lobe (ATL) was associated with trait respect. **(A)** Sagittal view of the left ATL whose GMV was negatively correlated with the score of the trait respect (*p* < 0.05, voxel-based FWE correction within the left ATL ROI). **(B)** A scatter plot showing the negative correlation between the score of the trait respect and the GMV of the peak voxel in the region in the left ATL associated with trait respect.

First, we analyzed the relationship between the score of trait respect and the GMV in the left ATL to identify brain regions associated with trait respect. We found a significant negative relationship of the GMV with the score of trait respect (voxel-level SVC-*p* < 0.05; Figures 1A,B and Table 1). There were no regions whose GMV was positively associated with the score reflecting trait respect. Notably, none of scores of subordinate categories of trait respect, including warm respect, worship, and awe, showed significant relationships with the GMV in the left ATL. We used the region that showed a negative relationship with the score reflecting trait respect as the trait respect-related region in the subsequent analysis.

**TABLE 1:** Negative relationship between the GMV and trait respect in a region in the left ATL.

Region	Hemisphere	Peak voxel in MNI space			*Z*-value	Number of voxels
		*x*	*y*	*z*		
ATL	Left	−36	0	−36	3.67	61

*In SVC analysis, voxel-based FWE correction was applied within the ROI for the left ATL. The statistical significance threshold was set at p < 0.05. MNI, Montreal Neurological Institute; ATL, anterior temporal lobe; SVC, small volume correction; FWE, family wise error; ROI, region of interest.*

Next, we analyzed the relationship between the GMV of the trait respect-related region and each subscale of trait empathy. Only the score of empathic concern showed a significant negative relationship with the GMV of the trait respect-related region (voxel-level SVC-*p* < 0.05; Table 2). As the score of trait respect was positively correlated with the scores of all four subscales of trait empathy (*p* < 0.001, two-sided test, Table 3), the relationship between empathic concern and reduced GMV was not merely due to the correlation between the score of trait respect and the score of empathic concern.

**TABLE 2:** A negative relationship between the GMV and empathic concern in a region in the left ATL.

Region	Hemisphere	Peak voxel in MNI space			*Z*-value	Number of voxels
		*x*	*y*	*z*		
ATL	Left	-36	2	-36	2.38	6

*In SVC analysis, voxel-based FWE correction was applied within the ROI where the brain region was associated with trait respect. The statistical significance threshold was set at p <0.05. MNI, Montreal Neurological Institute; ATL, anterior temporal lobe; SVC, small volume correction; FWE, family wise error; ROI, region of interest.*

**TABLE 3:** Correlation between trait respect and four subscales of trait empathy (*N* = 142).

	Perspective-taking	Fantasy	Empathic concern	Personal distress
Trait respect	0.29***	0.32***	0.41***	0.41***

***p < 0.001.

Since we investigated both male and female participants with a wide age range, we assessed the influence of sex and age on the score reflecting trait respect, the score reflecting empathic concern, and the GMV of trait respect-related regions. There was sex difference in the GMV (Table 4). The age was negatively correlated with both trait respect and the GMV (Table 5). However, a partial correlation between trait respect and the GMV, with age as a controlling variable, was significant (*r* = −0.304, *p* < 0.001). Thus, the relationship between trait respect and the GMV was not spurious.

**TABLE 4:** Influences of sex on the score of trait respect, the score of empathic concern, the GMV in the trait respect-related region.

	Male mean value (standard deviation)	Female mean value (standard deviation)	*t*-value	*p*-value
Trait respect	4.249 (0.743)	4.391 (0.742)	−1.120	0.265
Empathic concern	2.956 (0.329)	2.943 (0.291)	0.253	0.800
The GMV in the trait respect-related region	0.509 (0.075)	0.456 (0.064)	4.359	<0.001***

Paired-t test was used to evaluate sex difference in the variables. The degree of freedom of t-value was 140. ***p < 0.001.

**TABLE 5:** Influences of age on trait respect, empathic concern, the GMV in the trait respect-related region.

	Correlation coefficient	*p*-value
Trait respect	−0.246	0.003**
Empathic concern	−0.068	0.424
The GMV in the trait respect-related region	−0.300	<0.001***

Pearson product-moment correlation coefficient was used to evaluate influence of age on the variables. **p < 0.01, ***p < 0.001.

**FIGURE 2:**
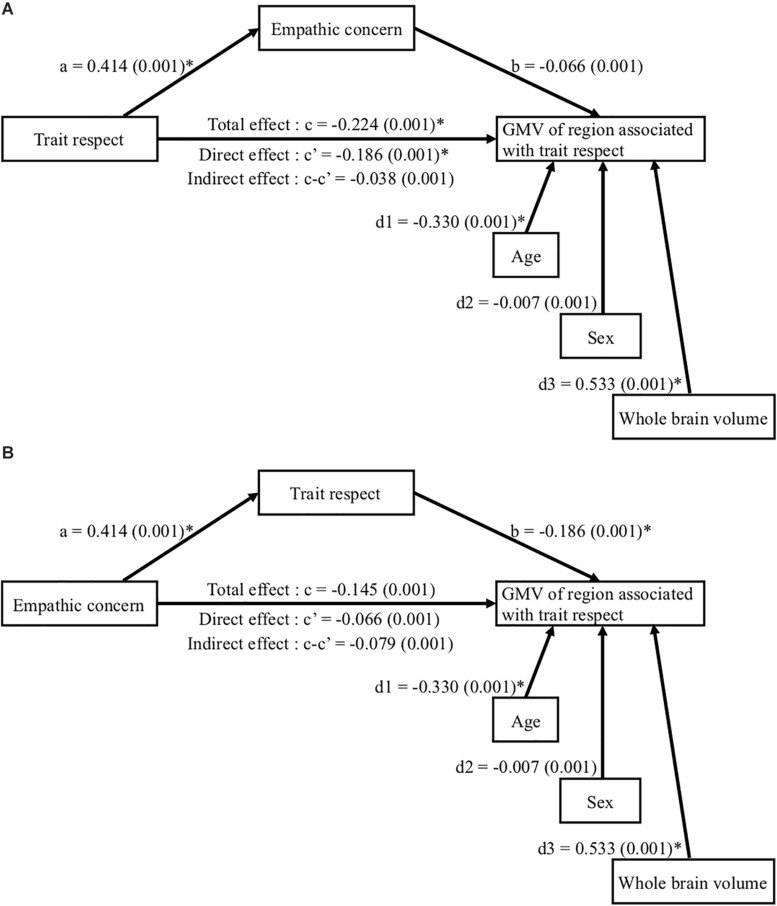
Direct and indirect effects of trait respect and empathic concern on the GMV in the trait respect-related region [peak voxel, MNI (−36 0 −36)]. Coefficients c, c’, and c-c’ indicate total, direct, and indirect effect on the GMV, respectively. **(A)** The mediation variable was empathic concern. Trait respect had a direct effect on the GMV. **(B)** The mediation variable was empathic concern. Standardized path coefficients (standard error) are shown for each path. ^∗^*p* < 0.05.

Finally, we conducted a mediation analysis to determine their direct and indirect effects on the GMV in the trait respect-related region. Age, sex, and whole brain volume were used to adjust the GMV. In Figure 2A, the mediator was empathic concern. There was a significant direct effect of trait respect on GMV and empathic concern. In Figure 2B, the mediator was trait respect. Although empathic concern had no direct effect on the GMV, empathic concern had an effect on trait respect and trait respect had an effect on the GMV.

## Discussion

Based on our previous findings regarding respect-related brain activity in the left ATL (Nakatani et al., 2019), we investigated whether trait respect is associated with morphological properties in the left ATL. We observed an association of trait respect with reduced GMV in the left ATL. Further, we examined whether the trait respect-related region was related to other social emotion categories with similar properties as respect. We observed an association of empathic concern with reduced GMV in the region. Moreover, mediation analysis revealed that trait respect had a direct effect on the GMV when empathic concern was a mediation variable.

GMV is positively associated with cognitive performance. For instance, expert taxi drivers in London present have increased GMV in task-related regions (Maguire et al., 2000) while training-related performance improvement is associated with increased GMV (Draganski et al., 2004; May, 2011; Woollett and Maguire, 2011). Moreover, non-use has been shown to reduce GMV (Langer et al., 2012). Therefore, an active involvement of the left ATL in respect indicates that trait respect should be associated with increased GMV. However, there have been reports of a negative relationship between GMV and cognitive performance. For example, expert chess players have reduced GMV in the caudate nucleus, precuneus, and occipitotemporal junction (Duan et al., 2012; Hänggi et al., 2014), which are involved in the perception of chess piece positions and quick move generation (Wan et al., 2011). Further, the trait of social emotions, including empathy and awe, are associated with reduced GMV in several brain regions (Banissy et al., 2012; Guan et al., 2019). Notably, we observed that trait respect and empathic concern were associated with reduced GMV in the left ATL. The association of trait respect and reduced GMV could be attributed to experience-dependent pruning of exuberant connections within the cortex. For example, early blind individuals without visual experience during the critical developmental period present with a thicker visual cortex than that in control individuals (Jiang et al., 2009). Social experiences in early childhood could cause stronger pruning of cortico-cortical connections and formation of social emotional traits.

We found that three subordinate categories of trait respect were not significantly associated with the GMV in the left ATL, which indicates that the morphological property of the ATL is associated not with a specific category but with a broad category of social emotions. This finding is consistent with the view that the ATL is involved in general conceptual knowledge (Rogers et al., 2004; Olson et al., 2007, 2013; Patterson et al., 2007; Zahn et al., 2007, 2009; Wong and Gallate, 2012). Moreover, the trait respect-related region was associated with empathic concern. Therefore, the region might be involved in other-oriented and affective aspect of social emotion.

This study has several limitations. First, the suggested experience-dependent pruning of exuberant connections within the cortex is just a speculation for explaining the relationship between trait respect and reduced GMV. However, this interpretation could be plausible with respect to brain plasticity and development. Second, we only focused only on the positive aspect of social emotions. Further, social emotions include negative aspects, such as envy, guilt, and shame. The morphological property of the region associated with trait respect could also contribute to other-oriented and affective aspects of negative social emotions. Further studies are needed to confirm this hypothesis. Nonetheless, our present and previous (Nakatani et al., 2019) studies indicate that the left ATL is a key brain region involved in respect.

## Conclusion

We assessed the morphological properties of the ATL in light of trait respect using VBM. There was a negative correlation between trait respect and the GMV in the left ATL; moreover, there was negative correlation between empathic concern and the GMV in the trait respect-related region. Our findings suggest that the left ATL is involved in other-oriented social emotions and contributes to a broad concept of respect.

## Data Availability Statement

The raw data supporting the conclusions of this article will be made available by the authors, without undue reservation, to any qualified researcher.

## Ethics Statement

The studies involving human participants were reviewed and approved by the Research Ethics Committee of the Graduate School of Arts and Sciences of the University of Tokyo. The participants provided their written informed consent to participate in this study.

## Author Contributions

KO organized the research project. YN conducted a pilot study. HN, YN, and SM designed the study. MA and YN conducted MRI scanning and the questionnaire survey. HN analyzed the data and drafted the manuscript. HN, YN, SM, MA, TN, and KO reviewed and edited the manuscript. All authors contributed to the article and approved the submitted version.

## Conflict of Interest

The authors declare that the research was conducted in the absence of any commercial or financial relationships that could be construed as a potential conflict of interest.

## Footnotes

1 http://atlas.brainnetome.org
